# Identification of sample annotation errors in gene expression datasets

**DOI:** 10.1007/s00204-015-1632-4

**Published:** 2015-11-25

**Authors:** Miriam Lohr, Birte Hellwig, Karolina Edlund, Johanna S. M. Mattsson, Johan Botling, Marcus Schmidt, Jan G. Hengstler, Patrick Micke, Jörg Rahnenführer

**Affiliations:** Department of Statistics, TU Dortmund University, Vogelpothsweg 87, 44227 Dortmund, Germany; Leibniz Research Centre for Working Environment and Human Factors (IfADo) at Dortmund TU, Dortmund, Germany; Department of Immunology, Genetics and Pathology, Uppsala University, Uppsala, Sweden; Department of Obstetrics and Gynecology, University Hospital, Mainz, Germany

**Keywords:** Gene expression, Microarray, Misannotation, Quality control, Male–female classifier

## Abstract

The comprehensive transcriptomic analysis of clinically annotated human tissue has found widespread use in oncology, cell biology, immunology, and toxicology. In cancer research, microarray-based gene expression profiling has successfully been applied to subclassify disease entities, predict therapy response, and identify cellular mechanisms. Public accessibility of raw data, together with corresponding information on clinicopathological parameters, offers the opportunity to reuse previously analyzed data and to gain statistical power by combining multiple datasets. However, results and conclusions obviously depend on the reliability of the available information. Here, we propose gene expression-based methods for identifying sample misannotations in public transcriptomic datasets. Sample mix-up can be detected by a classifier that differentiates between samples from male and female patients. Correlation analysis identifies multiple measurements of material from the same sample. The analysis of 45 datasets (including 4913 patients) revealed that erroneous sample annotation, affecting 40 % of the analyzed datasets, may be a more widespread phenomenon than previously thought. Removal of erroneously labelled samples may influence the results of the statistical evaluation in some datasets. Our methods may help to identify individual datasets that contain numerous discrepancies and could be routinely included into the statistical analysis of clinical gene expression data.

**Electronic supplementary material:**

The online version of this article (doi:10.1007/s00204-015-1632-4) contains supplementary material, which is available to authorized users.

## Introduction

The generation of large gene expression datasets presents a logistic challenge that extends from the initial procurement and storage of tissue samples, through laboratory procedures, to bioinformatic data processing and analysis. Although anticipated to be low, little is known about the actual frequency of sample mix-up during this multi-step process. The reasons for sample identity being swapped between individuals are diverse, and these events are difficult to pinpoint retrospectively with absolute certainty. In datasets with roughly balanced frequencies of male and female individuals, it can be assumed that approximately half of the mix-ups will result in sex mislabeling. These cases can be identified by assessment of genes with male- or female-specific expression. Other commonly annotated clinicopathological parameters, such as tumor stage, would also be affected by mislabeling, but the lack of genes that exhibit for instance a reliable stage-specific expression pattern makes the standardized assessment of these parameters unsuitable.

Few attempts have been made to systematically identify sample mix-ups in public gene expression datasets. The MixupMapper software (Westra et al. 2011) requires DNA sequence data (SNP) in addition to gene expression data. However, the majority of previous studies are based exclusively on gene expression data. Recent approaches use the expression of the X-chromosomal gene *XIST* and genes located on the Y chromosome for the discrimination between male and female samples in the analysis of single datasets. However, these methods are not generalizable because of the lack of normalization across datasets (‘t Hoen et al. 2013; Broman et al. 2015).

To gain insight into frequencies of sample annotation discrepancies in publicly available gene expression datasets, we established a male–female classifier based on gene expression array data. In addition, correlations between expression values for pairs of samples were assessed to identify multiple measurements of tissue from the same individual, as this represents an additional hypothetical source of inconsistencies with regard to sample annotation.

## Methods

In this investigation, 45 publicly available MIAME-compliant sample collections were included (see Tables 1, 2 for details), all with accessible gene expression array data and available information on male or female sex for each study subject. In total, the studies comprised 4913 patients (3034 females, 1879 males). Gene expression array data and information on male or female sex for each study subject were accessed from the Gene Expression Omnibus (GEO) or directly from the authors’ Web site (Edgar et al. 2002; Shedden et al. 2008; Bild et al. 2006). Only datasets using the AffymetrixGeneChip© HG-U133A or HG-U133 Plus 2.0 were included in this analysis.

**Table 1:** Overview of analyzed datasets

Type	Cohorts	Sample size (female/male)
Non-small cell lung cancer	GSE37745, Shedden, GSE31547, GSE29013, GSE14814, GSE4573, GSE31210, GSE19188, GSE31546, GSE10445	1338 (594/744)
Colon cancer	GSE33113, GSE12945, GSE31595, GSE4271, GSE1433, GSE17536, GSE17537	769 (358/411)
Other cancer	GSE5720, GSE4107, GSE42952, GSE34111, GSE31684	200 (64/136)
Non-cancer	GSE19027, GSE17913, GSE23343, GSE25462, GSE7821, GSE20950, GSE24427	408 (219/189)
Breast cancer	GSE11121, GSE2034, TRANSBIG (GSE7390/GSE6532), GSE16446, GSE20194, GSE20271, GSE22093, GSE23988	1373 (1373/0)
Ovarian cancer	Bild, GSE14764, GSE19829, GSE26712	426 (426/0)
Prostate cancer	GSE17951, GSE25136, GSE3325, GSE8218	399 (0/399)

Tissue collections and gene array datasets analyzed by the male–female classifier, if available identified by their Gene Expression Omnibus (GEO) Series (GSE) number

**Table 2:** Detailed description of analyzed datasets

Cohort	# Female	# Male	# Total	Type (disease or subject of study)
GSE37745	89	107	196	NSCLC
Shedden	220	223	443	NSCLC
GSE31547	36	14	50	NSCLC + controls
GSE29013	17	38	55	NSCLC
GSE14814	23	67	90	NSCLC
GSE4573	47	82	129	NSCLC
GSE31210	109	95	204	NSCLC
GSE19188	23	59	82	NSCLC
GSE31546	14	3	17	NSCLC
GSE10445	16	56	72	NSCLC
GSE4107	12	10	22	Colorectal cancer
GSE33113	48	42	90	Colorectal cancer
GSE31595	22	15	37	Colorectal cancer
GSE12945	28	34	62	Colorectal cancer
GSE14333	106	120	226	Colorectal cancer
GSE17536	81	96	177	Colorectal cancer
GSE17537	29	26	55	Colorectal cancer
GSE4271	32	68	100	Other cancer: glioma
GSE31684	25	68	93	Other cancer: bladder
GSE34111	6	24	30	Other cancer: gastrointestinal
GSE5720	24	30	54	Other cancer: 9 different tissues
GSE42952	9	14	23	Other cancer: pancreatic
GSE19027	11	48	59	Bronchial epithelium of (non-) smokers with and without lung cancer
GSE17913	38	40	78	Smoking
GSE23343	7	10	17	Insulin resistance/type 2 diabetes
GSE25462	28	22	50	Insulin resistance/type 2 diabetes
GSE7821	28	12	40	Healthy twins
GSE20950	27	12	39	Insulin resistance/obesity
GSE24427	80	45	125	Multiple sclerosis
GSE11121	200	0	200	Breast cancer
GSE2034	286	0	286	Breast cancer
TRANSBIG (GSE7390/GSE6532)	280	0	280	Breast cancer
GSE16446	114	0	114	Breast cancer; chemo response
GSE20194	247	0	247	Breast cancer; chemo response
GSE20271	139	0	139	Breast cancer; chemo response
GSE22093	47	0	47	Breast cancer; chemo response
GSE23988	60	0	60	Breast cancer; chemo response
Bild	133	0	133	Ovarian cancer
GSE14764	80	0	80	Ovarian cancer
GSE19829	28	0	28	Ovarian cancer
GSE26712	185	0	185	Ovarian cancer
GSE17951	0	153	153	Prostate cancer
GSE25136	0	79	79	Prostate cancer
GSE3325	0	19	19	Prostate cancer
GSE8218	0	148	148	Prostate cancer

Overview over the studied tissue collections and gene array data

To construct the classifier, we proceeded in three steps: 1. selection of probe sets with male- or female-specific expression, 2. dataset normalization to enable analysis of unlabelled cohorts and cohorts comprising only female or only male patients, and 3. combination of evidence from male- and female-specific probe sets into a final classifier that categorizes each sample as “correctly classified,” “misclassified,” or “unconfident.” In each step (1–3) a likelihood-based strategy was applied that ensures robustness against outliers (Algorithms 1–3 in Suppl. material).

**Fig. 1:**
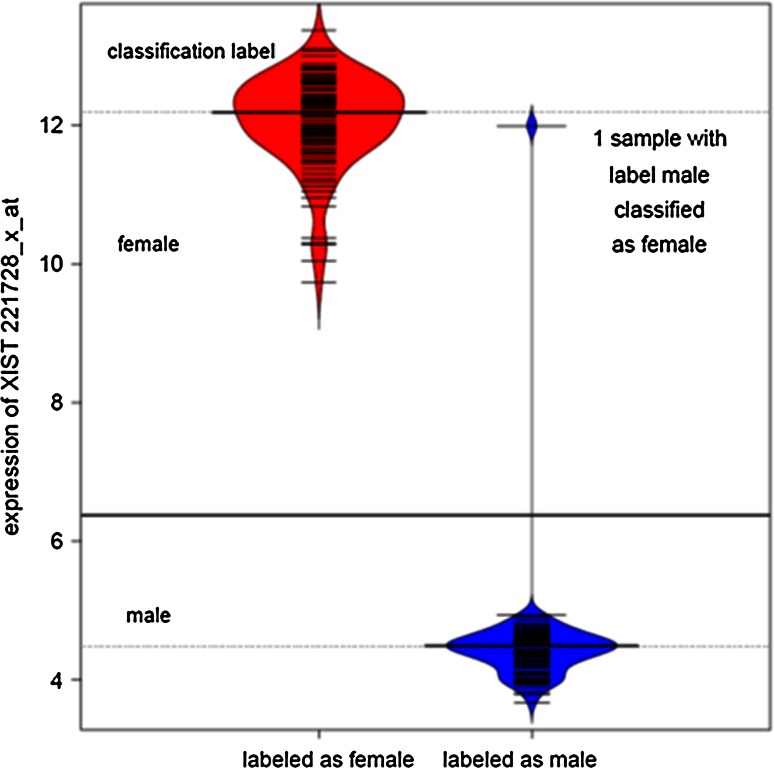
Differentiation between male and female samples by XIST expression. Bean plots of the expression values of probe set 221728_x_at (XIST) in the NSCLC cohort GSE31210. A clear separation between low expression values in males (*blue*) and high expression values in females (*red*) can be observed. One sample is mislabelled

The initial probe set selection was based on 10 publicly available non-small cell lung cancer (NSCLC) gene expression datasets analyzed on the AffymetrixGeneChip© HG-U133A or HG-U133 Plus 2.0 array (Suppl. material: Algorithm 1). For each sample, sex information and gene expression measurements for 22,277 probe sets were available. Only seven probe sets achieved median male–female classification accuracy above 75 % and only five above 90 %. The top four probe sets were included in the classifier (Table 3). Two of them map to the *XIST* gene (221728_x_at and 214218_s_at), located on the X chromosome, and the other two to *RPS4Y1* (201909_at) and *DDX3Y* (205000_at), respectively, both located on the Y chromosome. *XIST* is expressed from the inactive female X chromosome and silenced in men. This is illustrated in one NSCLC dataset (GSE31210), with high expression of *XIST* (221728_x_at) observed in all patients labelled as female (Fig. 1), but only in one sample labelled as male. Hence, this exception was clearly located in the female *XIST* expression range. *RPS4Y1* and *DDX3Y* showed the opposite behavior, with high expression values observed in male patients. *RPS4Y1* encodes a structurally conserved ribosomal protein with putative function during spermatogenesis (Lopes et al. 2010), whereas *DDX3Y* is primarily expressed in testis and is involved in germ-line translation control (Rauschendorf et al. 2011). Probe sets with low discriminating power were not included in the classifier.

**Table 3:** Probe sets included in the male–female classifier

Affymetrix ID	Gene	Chromosome	Cut point (99 % quantile)	Evidence (male/female)
221728_x_at	*XIST*	X	>0.389	Female
214218_s_at	*XIST*	X	>0.385	Female
201909_at	*RPS4Y1*	Y	>0.431	Male
205000_at	*DDX3Y*	Y	>0.276	Male

Probe sets included into the male–female classifier, with corresponding cut points for evidence whether a sample originates from a male or a female

**Fig. 2:**
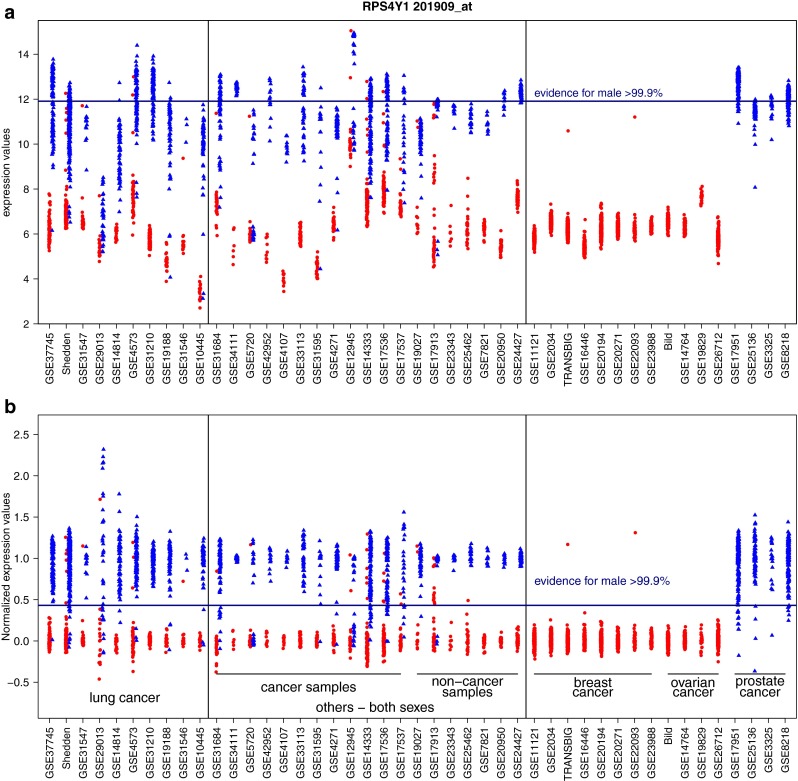
Improvement in comparability of cohorts by normalization. **a** Raw expression values of female (*red*) and male (*blue*) labelled samples set 201909_at (RPS4Y1) across all datasets. **b** The same cohorts after normalization. Specifically, two outliers in datasets TRANSBIG and GSE22093 indicate two breast cancer patients with high RPS4Y1 expression, feature clearly inconsistent with female sex

The expression levels of the four selected probe sets were evaluated in 35 additional datasets, including seven colon cancer, five other cancer, and seven non-cancer datasets containing samples from both male and female subjects, as well as eight breast cancer, four ovarian cancer, and four prostate cancer datasets. A plot of raw expression values for the probe set 201909_at (*RPS4Y1*) across all datasets showed high male–female classification accuracy per dataset, but large overall expression shifts between datasets (Fig. 2a). After normalizing expression values with a linear transformation to median values of 0 and 1 for the low and high expression groups, respectively (Suppl. material: Algorithm 2), expression levels were reliably comparable across cohorts (Fig. 2b).

In a final step, the four sex-specific probe sets were combined to categorize each sample as “correctly classified,” “misclassified,” or “unconfident” (Suppl. material: Algorithm 3). First, for each cohort and for each probe set, the expression values were clustered into two groups of low and high values and a normal distribution was fitted to the low expression group, estimating location and scale with robust measures (median and Rousseeuw–Croux estimator Q_n_ (Rousseeuw and Croux 1993)). Next, the expression value of the probe set for each sample was compared to the 99.9 % quantile of the fitted normal distribution. A value above this cut point is inconsistent with the typical range for the low expression group and thus provides strong evidence that the corresponding sample belongs to the high expression group. For each individual sample, a female-evidence score was then defined for each of the two *XIST* probe sets. As high *XIST* expression is inconsistent with male sex, the female-evidence score was set to 1 if the corresponding *XIST* expression value was above the cut point. Analogously, for *DDX3Y* and *RPS4Y1*, respectively, a male-evidence score was set to 1 if the expression value of the probe set was above the corresponding cut point. Taking the evidence scores of all four probe sets into account, a sample was classified as male if at least one male-evidence score was 1 and both female-evidence scores were 0. Vice versa, a sample was classified as female if at least one female-evidence score was 1 and both male-evidence scores were 0. Finally, the new classifications were compared to the original sex annotations, categorizing each sample as “correctly classified,” “misclassified,” or “unconfident.” Samples with both at least one positive female-evidence score and at least one positive male-evidence score, or with no positive evidence score, were classified as “unconfident.”

## Results and discussion

**Fig. 3:**
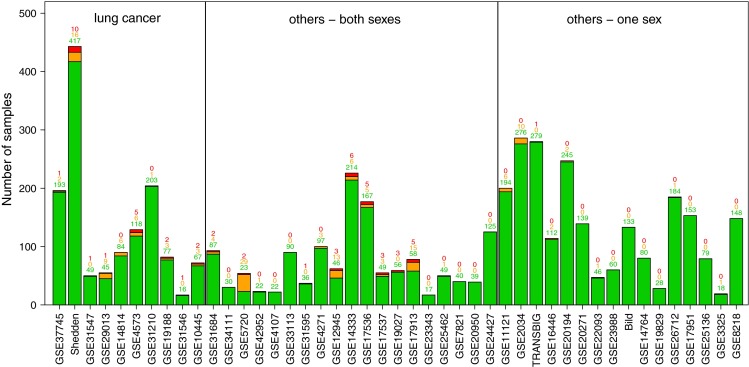
Application of the male–female classifier. Application of the male–female classifier to all cohorts, cohorts grouped by caner type. *Green* “correctly classified,” *red* “misclassified,” and *orange* “unconfident” samples

The male–female classifier was applied to all 45 cohorts, categorizing 4913 patients (3034 females, 1879 males) (Fig. 3). In total 54 patients (1.1 %) were categorized as “misclassified” and 149 (3.0 %) were labelled “unconfident.” The direction of sex mislabeling was nearly balanced, with 29 female samples mislabeled as male and 25 male samples mislabeled as female. Overall, in 18 of the 45 cohorts (40 %) at least one “misclassified” sample was detected. The proportion of “correctly classified” samples was 100 % in 15 cohorts, below 90 % in five cohorts, and in between for the remaining 25 cohorts. Note that these numbers are probably overoptimistic, as 16 cohorts included in the study consisted of breast, ovarian, or prostate cancer patients, with lower risk of sex mislabeling. Still, one breast cancer patient in the cohort TRANSBIG (comprising node-negative untreated patients of GSE7390 and GSE6532) was classified as male (Fig. 3).

**Fig. 4:**
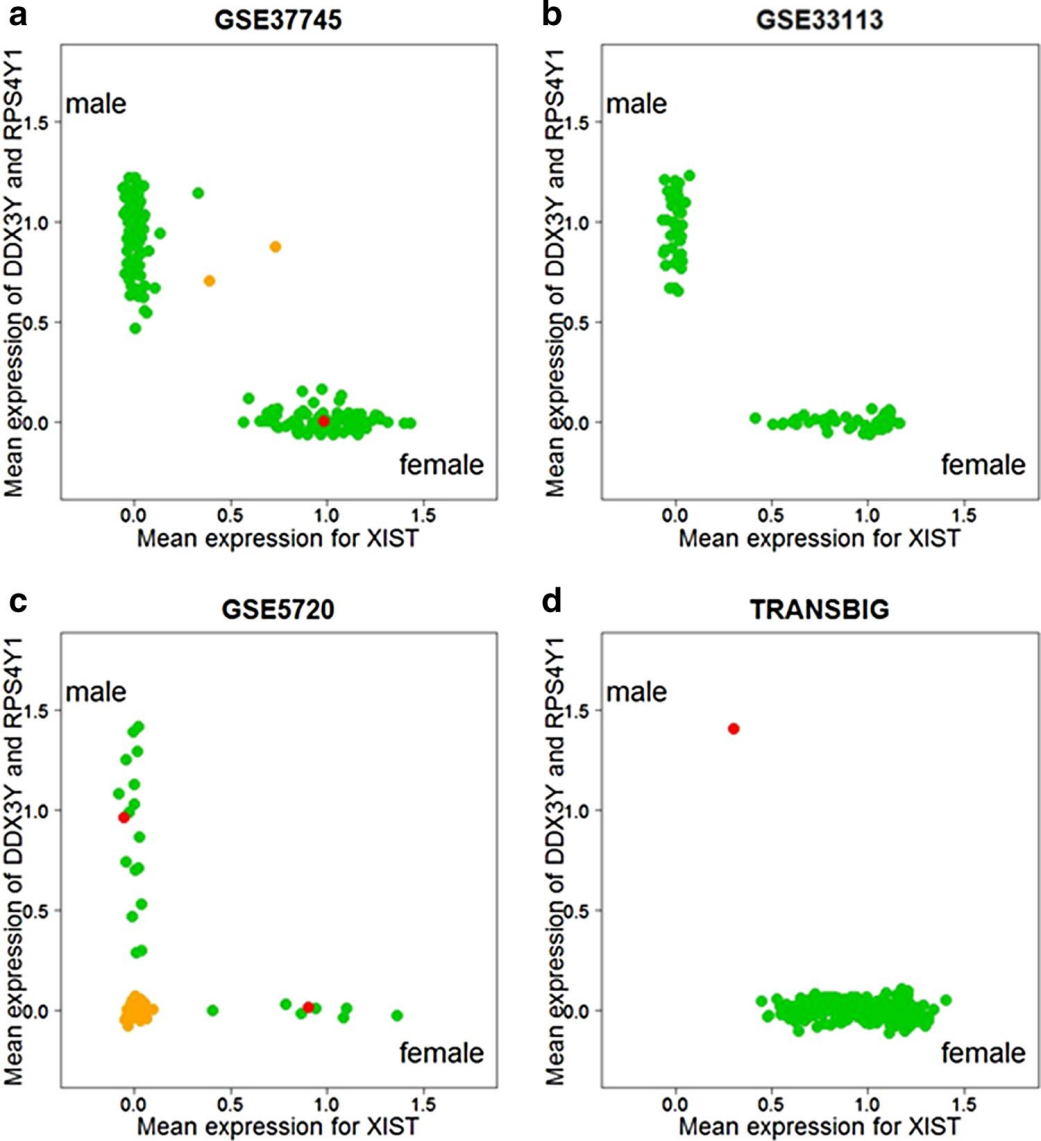
Visualization of the male–female classifier with mean expression values of the two prove sets for XIST on the *x*-axis and DDX3Y and RPS4Y1 on the *y*-axis. The points represent individual patients. The point clouds on the left and are characteristic for males and females, respectively. *Colors* indicate classification accuracy samples. *Green* “correctly classified,” *red* “misclassified,” and *orange* “unconfident.” **a** Results for the Uppsala cohort (GSE37745): One female patients clearly mislabeled as male, and two samples are labeled “unconfident.” **b** Results for GSE33113 with clear discrimination between males and females and no sex misannotations. **c** Results for GSE5720 with two misclassified samples and large number of samples classified as “unconfident.” **d** Results for a breast cancer dataset (TRANSBIG) with one male patient assigned to the category “misclassified”

The prevalence of sample identity inconsistencies in public data repositories can be anticipated to be at least twice as high as indicated by the male–female classifier, as mix-up may occur also between samples from individuals of the same sex. To visualize the expression-based sex assignment per cohort, we plotted mean normalized expression values of the two X-chromosomal probe sets and the two Y-chromosomal probe sets against each other (Fig. 4). For most cohorts, two clearly distinguishable groups representing males and females can be recognized, and category assignment by visual inspection is well in agreement with our likelihood-based classifier.

A further error that may occur during tissue processing is sample duplication. The same sample may be analyzed twice and the duplicate is erroneously labelled with the identification number of another patient. To identify such duplications, a correlation-based analysis strategy was applied. For each cohort, the 1000 probe sets with highest variance across all samples were selected and Pearson correlation coefficients between all pairs of samples in the cohort were calculated. The largest distance between all ordered values of correlations was identified to distinguish between duplicated measurements and pairs of measurements from different samples. In 15 of the 45 cohorts at least one duplicate was identified. In total 32 duplicates were detected. Comparing these duplicates with the results from the male–female classifier, nine of the 54 “misclassified” assignments (16.7 %) could be explained by duplicated measurements.

The general impact of misannotated samples on gene expression is difficult to assess. To illustrate the relevance of misannotations in gene expression studies, we re-analyzed six lung cancer cohorts with available survival times. Prognostic relevance of a gene was determined by fitting a univariate Cox model (Cox 1972) to its expression values. The number of significant genes (*p* value <0.01; not FDR-adjusted) was first calculated for the original datasets. Removing all unambiguously misannotated samples from the six datasets with misannotations, 12–53 % of the previously significant genes were not significant any more. In contrast, using only the reduced number of samples, the number of newly discovered genes was in the range of 9–39 % of the original number of significant genes (Table 4).

**Table 4:** Results of univariate Cox models

Dataset	No. of patients	No. of misannotations and duplications	No. of significant genes (original scenario)	Percentage of genes no longer significant after removal of the misannotated samples	Percentage of genes newly significant after removal of the misannotated samples
GSE37745	196	3	450	12.22	14.00
Shedden	443	14	1354	15.66	8.79
GSE29013	55	1	419	15.51	14.32
GSE4573	129	5	189	26.63	38.62
GSE31547	50	1	318	50.51	23.27
GSE19188	82	8	190	53.16	34,374

Results of univariate Cox models for six NSCLC datasets. Comparison between significance genes (*p* < 0.01) identified in the original cohort and significance genes identified in the reduced cohort after removal of misannotated and duplicated samples

To elucidate the reason behind the sample mislabeling observed in our own non-small cell lung cancer cohort (GSE37745), one patient annotated as male in the original records and assigned as female by our classifier was re-analyzed. First, new DNA and RNA samples were prepared from the original biobanked tissue specimen. Male sex was then confirmed based on the analysis of STR marker distribution using the AmpFLSTR^®^ Identifiler^®^ PCR Amplification Kit according to the manufacturer’s instructions (Applied Biosystems, Foster City, USA), suggesting that sample mix-up in this case did not occur during sample collection and biobanking procedures. Subsequently, the gene expression array analysis was repeated for the misclassified sample and for five additional control samples from the previously analyzed cohort. The pairwise correlation between the new and old misclassified sample was only 0.464, strongly indicating that these two samples were derived from different individuals. In contrast, a striking correlation of 0.993 was detected between the misclassified sample and a sample from one other female patient in the previously analyzed cohort. The high correlation suggests that the mRNA sample from one female patient erroneously had been measured twice in the previous analysis. A second duplicated measurement was detected, with correlation 0.990 between the expression values of two patients with sex label male. In contrast, all correlations of the repeated control samples with the corresponding original measurements were high (correlation coefficients: 0.910–0.987).

The rapidly increasing number of newly published results of microarray and RNA-seq experiments reveals that genome-wide expression data play an important role in translational research (Petermann et al. 2007; Verhaak et al. 2013). Therefore, quality control for gene expression measurements and clinical information on samples should be performed routinely before analyzing the data. Retrospective identification of misannotated samples is possible by a classifier-based computational strategy together with correlation analysis. In 18 of 45 cohorts analyzed at least one “misclassified” sample was detected. The easy-to-use classifier presented here, combined with correlation analysis to detect samples erroneously measured multiple times, helps to identify individual datasets that contain numerous discrepancies. Re-evaluation of gene expression array data demonstrated that sample mislabeling may have a considerable impact on the output of the statistical evaluation and allows inferences on the accuracy of biobanking. In conclusion, methods for identifying sample misannotations should be routinely included into the statistical analysis of clinical gene expression data.

## Electronic supplementary material

Supplementary material 1 (DOCX 54 kb)
