# Supplementary material for: Identification of sample annotation errors in gene expression datasets
Source: Arch Toxicol. 2015 Nov 25;89(12):2265–72. doi: 10.1007/s00204-015-1632-4 (PMC4673097; doi:10.1007/s00204-015-1632-4)
Supplement: Supplementary file 1 — Supplementary material 1 (DOCX 54 kb) [file 204_2015_1632_MOESM1_ESM.docx]

Description of the algorithms used for building and applying the male-female classifier described in

**Miriam Lohr, Birte Hellwig, Karolina Edlund, Johanna S.M. Mattsson, Johan Botling,
Marcus Schmidt, Jan G. Hengstler, Patrick Micke, Jörg Rahnenführer:**

*Identification of sample annotation errors in gene expression datasets*

Overview:

1. Algorithm for selection of variables (genes, probe sets) for male-female classification
2. Algorithm for normalization of datasets
3. Algorithm for the male-female classifier

**Algorithm 1: Selection of variables (genes, probe sets) for male-female classification**

**Input:**

- Raw expression values for $l$ cohorts (training datasets) for a list of candidate genes
- Sex annotation for every sample (male/female)
- Cutoff $q$ for probability for evidence whether a sample originates from a male or a female
- Cutoff $c$ for median classification accuracy per gene

**Output:**

List of genes for classifier with classification accuracy above cutoff $c$.

**Algorithm:**

All steps are carried out for each gene separately. First carry out steps 1-4 for every dataset $\left( i=1,\ldots,l \right)$ separately, then steps 5-6.

1. Estimate location of the values for males and for females, respectively, with the robust estimate median.
2. Call the smaller median $m_{0}$ and the larger median $m_{1}$, and estimate the scale with the robust Rousseeuw-Croux estimator $Q_{n,0}$ of the lower expression values.
3. Assign a sex-specific Gaussian distribution $f_{0}$ to the low expression values, with parameters $\mu_{0}=m_{0}$ and $\sigma_{0}^{2}=\left( 2.22Q_{n,0} \right)^{2}$.
4. Calculate the $q$ quantile of the distribution $f_{0}$ and classify a sample to the group with larger values if its expression value is above this cut point. Count the fraction of correctly classified samples, where the label is male (female) and where the corresponding expression value belongs to the expression values of the male (female) group.
5. Compute the median of correctly classified samples across all datasets and call it *median classification accuracy*.
6. If the *median classification accuracy* is above the cutoff *c*, include the corresponding gene in the male-female classifier.

**Algorithm 2: Normalization of values across cohorts**

**Input:**

- Raw expression values for a set of cohorts and for a specific gene
- Assignment of cohorts to training datasets and test datasets
- Sex information (if cohort belongs to training datasets)

**Output:**

Normalized expression values with group medians 0 and 1 for sex-specific subgroups, for every cohort.

**Algorithm:**

If the samples belong to both sexes, proceed as follows:

1. Calculate sex-specific location measures.
2. For a training dataset compute sex-specific medians and call the smaller value $m_{0}$ and the larger value$m_{1}$.
3. For a test dataset (or if no sex assignment is available) cluster all values with the
   k-means algorithm into 2 groups, choose as starting values the 25% and 75% quantile of all values. Call the smaller value of the two resulting cluster centers $m_{0}$ and the larger value$m_{1}$.
4. Calculate normalized expression values $\tilde{x}$ from original values x by $\tilde{x}=\frac{x-m_{0}}{m_{1}-m_{0}}$.

If all samples belong to one sex only (e.g. only females in a breast cancer dataset or only males in a prostate cancer dataset) proceed as follows:

The algorithm is described for a dataset $d$of females, for males proceed analogously.

1. Calculate the normalized expression values for all training datasets as described in **Step 1**. Then estimate a typical variation for females as the value of $Q_{n,\text{female}}$, applied to the set of all normalized values of females across all training datasets.
2. Estimate location and scale of the values in $d$ with the robust estimates $m_{d}=\mathrm{median}_{d}$ and $Q_{n,d}$.
3. Calculate normalized expression values $\tilde{x}$ from original values $x$ in $d$ by $\tilde{x}=\frac{x-m_{d}}{Q_{n,d}} Q_{n,\text{female}}$.
4. Add 1 to the normalized values if females correspond to the group with larger values, for the considered gene.

**Algorithm 3: Male-female classifier**

**Input**:

- Normalized expression values $\tilde{x}$ for one cohort $d$ for a list of $p$ genes, as a result of Algorithm 2
- Normalized expression values with group medians 0 and 1 for sex-specific subgroups for training datasets
- Chromosome allocation of the $p$ genes (located on X or on Y chromosome)
- $q$ Quantile $q_{d}$ of the estimated distribution

**Output:**

Classification of all samples of the cohort $d$ as *correctly classified*, *misclassified*, or *unconfident*.

**Algorithm:**

First carry out steps 1-2 for each of the $p$ genes separately, then proceed with step 3.

Steps 1-2 are described for a gene located on the X chromosome. For genes on the Y chromosome proceed in the same way, but with *female* replaced by *male* and *male* replaced by *female* in all steps.

1. Assign a sex-specific Gaussian distribution $f_{0}$ to the normalized expression values for males (as in Step 3 of Algorithm 1), with parameters $\mu_{0}=0$ and $\sigma_{0}^{2}=\left( 2.22Q_{n.0} \right)^{2}$, the latter based on all values that correspond to samples clustered to the male group, from all training datasets.
2. Compare each normalized expression value $\tilde{x}$ with the $q$ quantile of the estimated distribution $q_{d}$, and set the female evidence score to 1, if the normalized expression value $\tilde{x}$ is above the quantile $q_{d}$, and to 0 otherwise.
3. Classify a sample as
   - *correctly classified*, if the sample was labeled as *female*, at least one *female evidence score* is 1, and all *male evidence scores* are 0; or analogously for samples originally labeled as male.
   - *misclassified*, if the sample was labeled as *female*, at least one *male evidence score* is 1, and all *female evidence scores* are 0; or analogously for samples originally labeled as male.
   - *unconfident*, in all other cases.
